# Sparse Component Analysis (SCA) Based on Adaptive Time—Frequency Thresholding for Underdetermined Blind Source Separation (UBSS)

**DOI:** 10.3390/s23042060

**Published:** 2023-02-11

**Authors:** Norsalina Hassan, Dzati Athiar Ramli

**Affiliations:** 1 Department of Electrical Engineering, Politeknik Seberang Perai, Jalan Permatang Pauh, Bukit Mertajam 13700, Pulau Pinang, Malaysia; 2 School of Electrical & Electronic Engineering, Universiti Sains Malaysia, Nibong Tebal 14300, Pulau Pinang, Malaysia

**Keywords:** underdetermined blind source separation, sparse component analysis, mixing matrix estimation

## Abstract

Blind source separation (BSS) recovers source signals from observations without knowing the mixing process or source signals. Underdetermined blind source separation (UBSS) occurs when there are fewer mixes than source signals. Sparse component analysis (SCA) is a general UBSS solution that benefits from sparse source signals which consists of (1) mixing matrix estimation and (2) source recovery estimation. The first stage of SCA is crucial, as it will have an impact on the recovery of the source. Single-source points (SSPs) were detected and clustered during the process of mixing matrix estimation. Adaptive time–frequency thresholding (ATFT) was introduced to increase the accuracy of the mixing matrix estimations. ATFT only used significant TF coefficients to detect the SSPs. After identifying the SSPs, hierarchical clustering approximates the mixing matrix. The second stage of SCA estimated the source recovery using least squares methods. The mixing matrix and source recovery estimations were evaluated using the error rate and mean squared error (MSE) metrics. The experimental results on four bioacoustics signals using ATFT demonstrated that the proposed technique outperformed the baseline method, Zhen’s method, and three state-of-the-art methods over a wide range of signal-to-noise ratio (SNR) ranges while consuming less time.

## 1. Introduction

When the original source signals of interest cannot be recorded directly and only a combination of mixtures is available, a source separation problem will exist. The objective of the source separation problem is to isolate the original signals from a combination of several source signals. Blind source separation (BSS) is a typical method used for source separation in the signal processing research community and it has gained considerable attention [1,2,3]. Recently, BSS theory has been refined and enhanced. In selecting the proper BSS approach, the link between the number of observations (sensors) and the number of underlying sources is of paramount importance. BSS is categorised as determined BSS (M = N), overdetermined BSS (M > N) and underdetermined BSS (UBSS) (M < N), where M represents the number of observations (sensors) and N represents the number of sources [4]. Despite being a trickier problem than the other two branches of BSS problems, UBSS is the most popular one because it is the most suitable BSS to suit practical applications, especially when the number of sensors is less than the number of sources [5].

UBSS has garnered substantial scholarly interest, and numerous solutions to this issue have been offered [6,7,8,9,10]. UBSS is classified into two classes, namely, statistic characteristic and sparsity [11]. Sparsity is a potent regulatory concept for reducing the number of possible solutions or locating a more realistic solution. In a given representation, a signal is considered sparse if a small number of coefficients can adequately explain the majority of its characteristics. If the source signals are sparse, it is easy to identify the mixing mechanism. The sparse component analysis (SCA) methodology has emerged as a crucial method for dealing with UBSS because it uses the sparseness of nonstationary signals [12]. UBSS based on SCA is divided into two primary stages: the estimation of the mixing matrix and the estimation of the source signal [13]. Since the exact estimation of the mixing matrix is the foundation of signal recovery, the first step must be precise. Sparsity-based practices focus on extracting the non-negative sources. Much work has been carried out on what is called SCA [14,15,16,17,18]. Several techniques for obtaining sparsity in the transform domain have been developed thus far, including the short-time Fourier Transform (STFT) and the wavelet packet transform [19,20,21,22,23]. UBSS techniques are highly dependent on the sparsity of the source signal. If the signal in the TF plane is not sufficiently sparse or if noises exist after the transformation, there will be some outliers present. To solve either of these issues and boost the estimated performance of the mixing matrix, several estimation techniques based on the detection of single-source points (SSPs) have been developed [16,24,25,26,27,28,29,30]. 

The authors of [25] introduced the concept of SSP detection. The authors introduced the time–frequency (TF) ratio of the mixtures (TIFROM) to further relax the assumption of a TF disjoint. This strategy provided some overlaps between the source of the TF representations. The identification of SSPs using the ratio of the TF transforms was also reported in [30]. By proposing an STFT-based technique for estimating the mixing matrix, [24] demonstrated that a TF point is an SSP when the absolute direction of the real and the imaginary parts of the TF vectors of the mixed signals are the same. The same procedure of detecting the SSPs can be found in [13]. For a time-delayed mixing matrix with a single-source point, ref. [27] showed that the time-delayed mixing model of the UBSS problem works well with the single-source identification-based mixing matrix estimation strategy. The authors of [26] utilised the single-source detection (SSD) algorithm that recognises the TF points occupied by a single source for each source. An SSP identification technique based on the transformation matrix was proposed by [28]. The number of source signals was then identified by looking for the maximum value of the potential function. However, the existence of noise in the system had a considerable impact on the selection of the peak values. Most of the SSP-based UBSS algorithms ignored the link between SSPs, resulting in low SSP identification accuracy, especially in noisy cases. The authors of [16] proposed recognising SSPs by using sparse coding, which accounts for the linear correlations between SSPs. Consequently, even at a low signal-to-noise ratio (SNR), this approach has been observed to be capable of delivering exceptional mixing matrix estimation performance. Table 1 shows a summary of the research related to SSP detection.

The method in [16] used a predetermined parameter to select the STFT coefficients before detecting the SSPs to estimate the mixing matrix. Choosing the right parameter is difficult since incorrect choices will increase the errors in the estimated source. Consequently, it remains challenging to identify SSPs reliably and effectively. To estimate the mixing matrix, this research constructed the first stage of UBSS by using SCA and a technique prior to SSP detection. The work was inspired by the following aims: (1) how to enhance the accuracy of the mixing matrix estimation and (2) how to reduce the computational time (time cost) in estimating the mixing matrix. The authors of [30] proved that only a minimal number of TF coefficients is enough to fully estimate the mixing matrix before identifying single source points. The TF selection factor is also able to increase the level of accuracy and boost the computational efficiency. From this perspective, in comparison with the method in [16], an adaptive algorithm for selecting the significant coefficient before it can be used as an input to detect the SSPs was proposed in this study. 

The goal of this work was to propose an adaptive approach for selecting meaningful data from TF mixtures that facilitates better identification of SSPs. The study focused on increasing the accuracy and decreasing the time costs of estimating the mixing matrix as well as enhancing the quality of source signal separation. After identifying the SSPs in the TF plane during the estimation of the mixing matrix, these points are clustered by hierarchical clustering to estimate the mixing matrix. The second stage of SCA employed the least squares method to carry out the source recovery estimation.

The remainder of this article is organised as follows. Section 2 describes the generation of mixed signals. The proposed method is introduced in Section 3. Section 4 discusses the source recovery method. Section 5 analyses the performance of the proposed method, and the conclusions are given in Section 5.

## 2. The UBSS–SCA-Based Method

**Figure 1:**
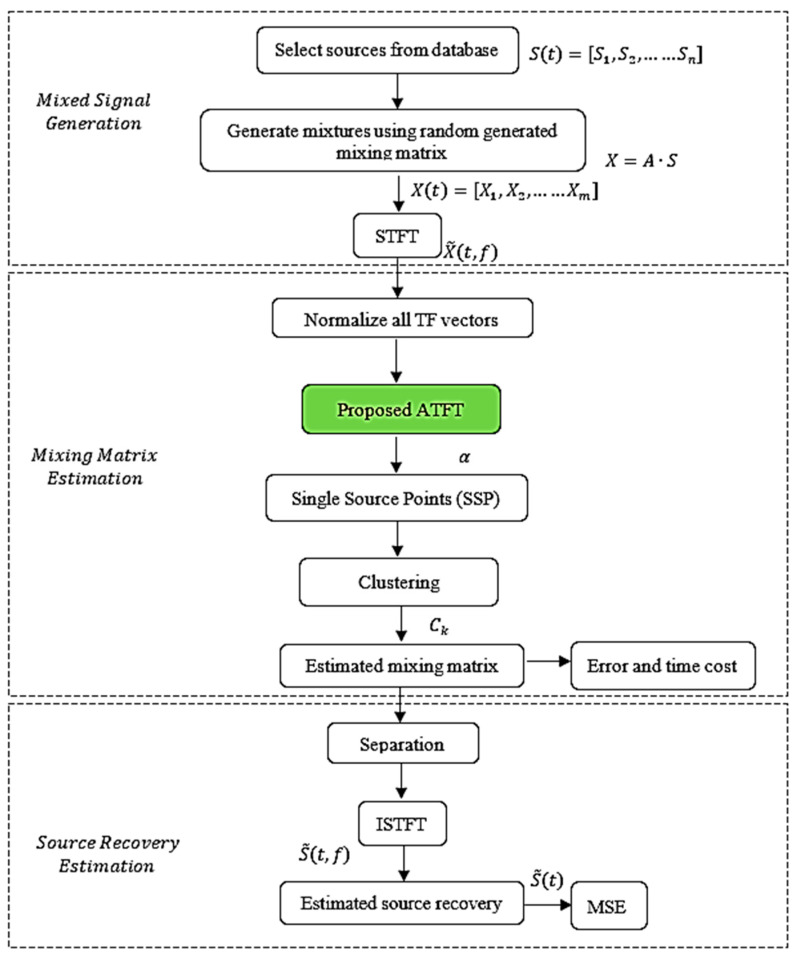
General flow chart of the entire process.

The UBSS approach based on SCA is made up of two phases. The initial step of the procedure is to estimate the mixing matrix, followed by estimating the source signal. The procedure starts with mixed signal generation. The flowchart of the entire process is shown in Figure 1, with the proposed technique noted in the green box.

## 3. Mixed Signal Generation

This study considered an underdetermined instantaneous mixed model where the mixed signal in the time domain can be expressed as

(1) $$X\left(t\right)=AS(t)$$

where $X\left(t\right)={\left[X_{1}(t),X_{2}(t),\ldots ,X_{M}(t)\right]}^{T}$ represents the *M*-many mixed signals received by the sensors at the receiving end, $A={\left[a_{1},a_{2},\ldots ,a_{N}\right]}^{}$ is the mixing matrix, the ith column is called the steering vector corresponding to the ith source, $S\left(t\right)={\left[S_{1}(t),S_{2}(t),\ldots ,S_{N}(t)\right]}^{T}$ represents the *M*-many sources, *T* is the instant time and ${}^{T}$ represents the transpose operation. STFT was used as the sparse method to demonstrate the mechanism of SCA processing. In STFT, sparsity indicates that in every particular time–frequency (TF) bin, the energy of only one of the speakers dominated. The mixed signals were made sparser by applying STFT to Equation (1). Both sides utilised STFT without noise for convenience. The transformed model (the TF domain) of the mixtures was obtained as Equation (2) as follows:

(2) $$\tilde{X}\left(t,f\right)=A\tilde{S}(t,f)$$

where $\tilde{X}\left(t,f\right)={\left[{\tilde{X}}_{1}(t,f),{\tilde{X}}_{2}(t,f),\ldots ,{\tilde{X}}_{M}(t,f)\right]}^{T}$, with ${\tilde{X}}_{i}\left(t,f\right)$ standing for the STFT coefficients of the ith mixture’s mixed signals in the fth frequency bin at time t, and $\tilde{S}\left(t,f\right)={\left[{\tilde{S}}_{1}(t,f),{\tilde{S}}_{2}(t,f),\ldots ,{\tilde{S}}_{N}(t,f)\right]}^{T}$, with ${\tilde{S}}_{j}\left(t,f\right)$ representing the STFT coefficients of the jth source signal in the fth frequency bin at time t.

The mixed signals in the time–frequency (TF) domain was used to estimate the mixing matrix. The mixing matrix estimation is an essential procedure in SCA, and it can be improved in two ways: single-source point (SSP) detection and clustering. In this work, an adaptive algorithm was applied before the SSP detection step to increase the accuracy and reduce the complexity. An SSP is a time–frequency point at which the energy of just one source is significant, while the energies of signals from other sources are either zero or very near to zero at that point. By locating the SSPs, the data clustering can be enhanced, allowing clustering algorithms to be able to estimate the mixing matrix more accurately.

## 4. Mixing Matrix Estimation

### 4.1. The Proposed Adaptive Time–Frequency Thresholding (ATFT) Method

**Figure 2:**
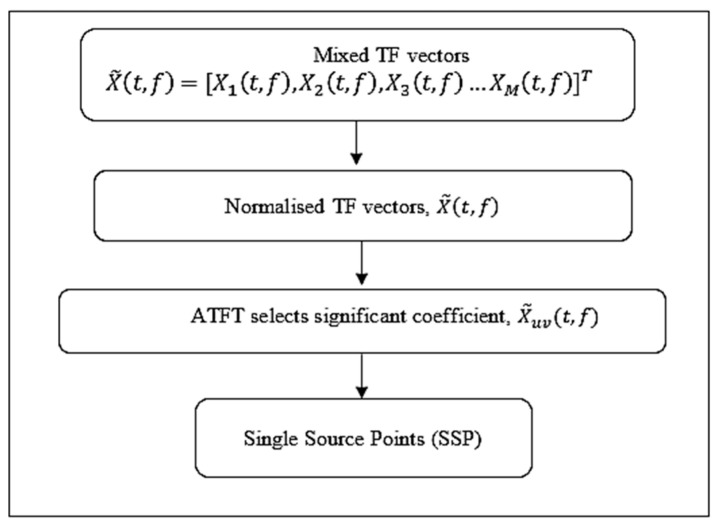
The flowchart of the proposed ATFT method.

Through the development of mixing matrix estimation, research has been carried out to find a new way to improve SCA. Estimating an unknown mixing matrix accurately is crucial to provide a sufficient condition for the recoverability of a source vector. Many different SSP detection strategies have been put forth as means of enhancing the accuracy of mixing matrix estimates. By extracting 1D subspaces from the whole collection of TF representation vectors of the observed mixed signals, ref. [16] provided a technique for identifying SSPs that took a pragmatic approach. To estimate the mixing matrix, it employed a fixed parameter to decide which STFT coefficients to use before looking for the SSPs. Only a few TF coefficients were found to be sufficient to fully estimate the mixing matrix before identifying single-source locations, as stated by [8]. However, there is another possible method of improving the accuracy and computing the efficiency, which is by using a TF selection factor. An algorithm that can adaptively determine the significant coefficients was proposed in this work to better estimate the mixing matrix. The primary contribution of this work was to formulate a method, which was called adaptive time–frequency thresholding (ATFT) and was able to reduce the dimensions of a mixture of TF vectors. This decrease was important so that the complexity was reduced, and a significant coefficient was selected accurately before it was used as an input to detect the single-source points (SSPs). The steps of the proposed ATFT method are shown in Figure 2.

According to Figure 2, the objective here is to select the significant coefficients of the TF vectors rather than blindly detecting the SSPs by using all the TF vectors. Through use of the proposed ATFT method, the significant TF vector was chosen by setting an adaptive threshold, α, to select the column with more energy than the given level. The threshold was calculated via the following steps: 

Step 1: The normalisation process was executed for each element in the TF vectors of the mixed signals would have a unit norm, using the following operation:

(3) $${TF}_{{norm}_{j}}=\sum_{i=1}^{j}{\left|{\tilde{X}}_{Mj}(t,f)\right|}^{2}$$

where M is the number of mixed signals and j=1,2,3…,D, and D is the dimension/number of elements in the array. Next, the mean of the norm of the results in Equation (3) was calculated and denoted as

(4) $$E({TF}_{{norm}_{j}})=\frac{1}{D}\sum_{j=1}^{D}{TF}_{{norm}_{j}}$$

Step 2: An adaptive threshold, α, was set to select the significant coefficient from the TF mixtures.

1. Let ${index}_{j}=[1,2,3,\ldots ,D]$.
2. The threshold of ATFT, α, is obtained from the norm of the result of all mixtures and was computed by
  (5) $$\alpha ={\left(\sum_{d=0}^{D-1}{\left|{TF}_{{norm}_{j}}\right|}^{2}\right)}^{1/2}/D$$

The threshold values varied for each different mixture and dataset. The selected column must meet the condition.

3. According to Equation (5), the thresholding operation is defined as
  (6) $${index}_{j}=\left\{\begin{array}{l} 1,{TF}_{{norm}_{j}}>\frac{E({TF}_{{norm}_{j}})}{\alpha }\\ 0,otherwise \end{array}\right.$$
  where j=1,2,3…,D. Here, ${ind}_{j}$ shows that only the mixture vectors with the norm that is greater than $\frac{E({TF}_{{norm}_{j}})}{\alpha }$ is used. This will determine which column of the TF vectors will be used for the following process.

Step 3: From Equation (6), the corresponding column of the TF mixture vectors will be selected as follows:

(7) $${\tilde{X}}_{yz}\left(t,f\right)=\left\{\begin{array}{l} {\tilde{X}}_{ij}\left(t,f\right){\mathrm{index}}_{j}=1\\ not\;select,otherwise \end{array}\right.$$

According to Equation (7), y=i, $j=1,2,3\ldots ,D,z=1,2,3\ldots ,\hat{D}$; $\hat{D}$ is the new dimension and ${\tilde{X}}_{yz}\left(t,f\right)$ represents the new TF vectors which are selected adaptively from the initial TF vectors in Equation (2) using Equation (5). The purposes of this step are to select the significant coefficients and reduce the computational burden. Only ${\tilde{X}}_{yz}\left(t,f\right)$ is used further for SSP detection and mixing matrix estimation. Selection of the column with a significant coefficient, the mixing matrix estimation can be improved.

### 4.2. Single Source Point (SSP) Detection

In this study, sparse coding was used to find and extract the SSPs from the ATFT method’s mixture vectors. Sparse coding is a technique for representing an input signal with a limited number of basic functions [31]. Sparse coding was applied for each ${\tilde{X}}_{yz}\left(t,f\right)$ to detect the SSPs. Two assumptions were made to estimate the mixing matrix, i.e., (A1) in the mixing matrix A, any column vector is linearly independent; (A2) there are some TF points (t,f) for each source $s_{i}^{\prime }$ in which only $s_{i}^{\prime }$ is dominant, $\left|{\tilde{S}}_{i}(t,f)\right|\geq \left|{\tilde{S}}_{j}(t,f)\right|\forall_{j}\neq i$ [19]. Based on these assumptions, for each source signal $S\left(t\right)$ at any TF point such as (u,v) where only one source is active, ${\tilde{S}}_{i}(u,v)$ is written as

(8) $$\tilde{X}\left(u,v\right)={\tilde{S}}_{i}(u,v)a_{i}$$

where (u,v) is an SSP corresponding to $S\left(t\right)$. Using the mixture vectors at these SSPs allows the estimation of the column vectors of the mixing matrix. At another TF point such as (φ,ω) with only a single active source, ${\tilde{S}}_{i}(\varphi ,\omega )$, for each source signal $S\left(t\right),$ Equation (9) is obtained as

(9) $$\tilde{X}\left(\varphi ,\omega \right)={\tilde{S}}_{i}(\varphi ,\omega )a_{i}$$

From Equations (8) and (9), both the SSPs, $\tilde{X}\left(u,v\right)$ and $\tilde{X}\left(\varphi ,\omega \right)$, are collinear with $a_{i}$ at $\left(u,v\right)$ and $\left(\varphi ,\omega \right)$ respectively. As validated by [16], if $\tilde{X}\left(u,v\right)$ and $\tilde{X}\left(\varphi ,\omega \right)$ are both SSPs corresponding to the same active sources, they can be linearly represented as

(10) $$\tilde{X}\left(u,v\right)=\tilde{X}\left(\varphi ,\omega \right)r$$

where r is a real number. The SSP can be represented as a one-dimensional (1D) subspace, and its identification can be viewed as the identification of the 1D subspaces in datasets of mixed signals using sparse coding techniques. To obtain the sparse coding solution, each mixture’s ATFT vectors can be written as a linear combination of other TF mixtures’ vectors, $\tilde{Y_{i}}$:

(11) $$\tilde{Y_{i}}=\tilde{Y}b_{i},\;s.t.\;b_{ii}=0$$

where $\tilde{Y}≜[y_{1},y_{2},\ldots ,y_{q}]$; $y_{1},y_{2},\ldots ,y_{q}$ form the set of all the mixtures’ ATFT vectors; $b_{i}≜[b_{i1},b_{i2},\ldots ,b_{iq}]$ is the coding coefficient vector and q is the number of TF points. The trivial approach of expressing a mixture TF vector by itself is eliminated by the constraint $b_{ii}=0$. Sparse coding attempts to find a solution, $b_{i}$, in which nonzero entries are a mixture of the TF vectors from the same subspace as $\tilde{Y_{i}}$; $b_{i}$ is the sparse representation of $\tilde{Y_{i}}$. If $b_{i}$ is sufficiently sparse, the solution to Equation (11) is acquired by optimising the following objective function:

(12) $$J\left(b_{i};\lambda \right)=\lambda {\left\|b_{i}\right\|}_{1}+\frac{1}{2}{\left\|{\tilde{Y}}_{i}-\tilde{Y}b_{i}\right\|}_{2}^{2}\;s.t.b_{ii}=0$$

where λ>0 is a regularisation parameter that controls the trade-off between good reconstruction and sparsity in the representation. The value of 0.001 was chosen for λ in this study. A column vector of $\tilde{Y}$ was extracted corresponding to the only nonzero element of b corresponding to one point of the 1D subspace of $\tilde{Y}.$ The $l_{1}$-norm solver from the MATLAB package was used, and this convex minimisation problem effectively solved Equation (12) by using the convex optimisation method. 

After obtaining the sparse coding solutions for each TF point, TF points with just one nonzero element were considered in the sparse coding coefficient vector to be SSPs. Following the identification of the SSPs, the next step was to estimate the mixing matrix. The detected SSPs consisted of the set of normalised column vectors of the mixing matrix denoted as Ω, as stated in Equation (13):

(13) $$\Omega =\{y_{1},y_{2},\ldots ,y_{N}\}$$

To aid the clustering analysis, the data must be normalised after the SSP detection process, which entails mapping the data points to the positive half-unit circle. The normalised SSPs then will be clustered by the clustering method. In summary, the proposed ATFT and SSP approaches are described in Algorithm 1.

**Algorithm 1:** Mixing matrix estimation procedure using the proposed ATFT method**Input**: The mixed signal, $\hat{X}\left(t\right)$.
$\hat{X}\left(t\right)$ is transformed from the time domain into the TF domain using STFT to produce $\tilde{X}\left(t,f\right)$.
Each element in the mixture vectors, $\tilde{X}\left(t,f\right)$, is normalised to have a unit norm, ${TF}_{{norm}_{j}}=\sum_{i=1}^{j}{\left|{\tilde{X}}_{Mj}(t,f)\right|}^{2}$ where j=1,2,3…,D. D is the dimension.
The mean of the unit norm, E$({TF}_{{norm}_{j}})=\frac{1}{D}\sum_{j=1}^{D}{TF}_{{norm}_{j}},is\;computed$.
The threshold α is used to select the significant coefficient.Let ${index}_{j}=[1,2,3,\ldots ,D]$
$${index}_{j}=\left\{\begin{array}{l} 1,{TF}_{{norm}_{j}}>\frac{E({TF}_{{norm}_{j}})}{\alpha }\\ 0,otherwise \end{array}\right.$$where j=1,2,3…,D. $\alpha =3{\left(\sum_{d=0}^{D-1}{\left|{TF}_{{norm}_{j}}\right|}^{2}\right)}^{1/2}/D$, and N=D${\tilde{X}}_{yz}\left(t,f\right)=\left\{\begin{array}{l} {\tilde{X}}_{ij}(t,f){index}_{j}=1\\ not\;select,otherwise \end{array}\right.$where u=i, $j=1,2,3\ldots ,D,v=1,2,3\ldots ,\hat{D}$. $\hat{D}$= new dimension
For each ${\tilde{X}}_{yz}\left(t,f\right)$, the sparse coding coefficients are computed by using $l_{1}$-norm optimisation.
The mixture of TF vectors with sparse coding coefficient vectors containing only one nonzero element is added into Ω.
The elements are normalised on Ω.
**Output**: Normalised Ω.

### 4.3. Clustering

The hierarchical clustering algorithm [24] was used to cluster and extract the SSPs vectors, $Ω=\{y_{1},y_{2},\ldots ,y_{N}\}$, followed by grouping its elements into n clusters. The initial cluster centre was set as $C=[c_{1},c_{2},\ldots ,c_{n}]$ and n represented the cluster’s centre. In addition, $1-\left|\cos\theta \right|$ was used as the distance measure, where cos⁡θ =${\tilde{X}}_{m}^{T}{\tilde{X}}_{n}/(\left\|{\tilde{X}}_{m}\right\|\left\|{\tilde{X}}_{m}\right\|)$ is the cosine of the angle between the mth and nth sample vectors ${\tilde{X}}_{m}$ and ${\tilde{X}}_{n}$ in $\tilde{X}$. The centroid of each cluster was identified after clustering to compute the column vectors of the mixing matrix. To further reduce the error of the mixing matrix estimation, outlier points were those that were far from the cluster’s mean direction and were eliminated. Points in the data that met the requirement $\left|{Ø}_{Q}\left(i\right)-\mu_{{Ø}_{Q}}\right|>{\epsilon \sigma }_{{Ø}_{Q}}$ were removed, where ${Ø}_{Q}\left(i\right)$ is the absolute direction of the $i_{th}$ sample in the $Q_{th}$ cluster and $\mu_{{Ø}_{Q}}$ is the mean of the absolute direction of the samples in the $Q_{th}$ cluster. The new cluster centre $C_{new}=[c_{new1},c_{new2},\ldots ,c_{newn}]$ was obtained via the mean value of each cluster. The procedure is summarised in Algorithm 2. 

**Algorithm 2:** The procedure of mixing matrix estimation using hierarchical clustering**Input:** The extracted SSP vectors, $Ω=\{y_{1},y_{2},\ldots ,y_{N}\}$.
The clustering method is applied to the extracted SSPs to group its elements into n clusters.The centres of these n clusters are calculated as the estimations for the columns of the mixing matrix to produce the estimated mixing matrix.The outliers are removed and $C_{new}$ is produced.
The centres of the clusters are calculated as the estimated mixing matrix, $\tilde{A}$.
**Output:** The estimated mixing matrix, $\tilde{A}$ is formed.

## 5. Source Recovery Estimation

To construct a sparse TF representation of the recovered sources, a sequence of least square problems was used to recover the source signals, minimising the error function by selecting the optimal $M\times \left(M-1\right)$ submatrix of $\tilde{A}$. Let A be a set composed of all $M\times \left(M-1\right)$ submatrices of the estimated mixing matrix, $\tilde{A}$; that is,

(14) $$A=\left\{A_{i}|A_{i}=[{\hat{a}}_{\theta_{1}},{\hat{a}}_{\theta_{1}},\ldots ,{\hat{a}}_{\theta_{M-1}}]\right\}$$

where the indices of these (M-1)-many nonzero elements are denoted as $\theta_{1},\theta_{2},\ldots ,\theta_{M-1}$ and A has $C_{N}^{\left(M-1\right)}$ elements, $i=1,2,\ldots ,C_{N}^{\left(M-1\right)}$. For each TF point $\tilde{X}\left(t,f\right)$, there is submatrix $A_{*}=[{\hat{a}}_{\emptyset_{1}},{\hat{a}}_{\emptyset_{2}},\ldots ,{\hat{a}}_{\emptyset_{M-1}}]$ in the set A that satisfies Equation (15):

(15) $$\tilde{X}(t,f)=A_{*}A_{*}^{†}\tilde{X}(t,f)$$

where $A_{*}^{†}$ is the pseudo-inverse of $A_{*}$. $A_{*}$ constructs an n-dimensional vector $S\left(t,f\right)$ by setting its jth element as

(16) $${\tilde{S}}_{j}\left(t,f\right)=\left\{\begin{array}{l} e_{i},if\;j=\emptyset_{i}\\ 0,otherwise \end{array}\right.$$

where $e={\left[e_{1},e_{2},\ldots ,e_{M-1}\right]}^{T}=A_{*}^{†}\tilde{X}\left(t,f\right),$ and $A_{*}$ is obtained from Equation (17):

(17) $$A_{*}=\arg\underset{A_{i}\in A}{\min}{\left\|\tilde{X}\left(t,f\right)-A_{i}A_{i}^{†}\tilde{X}(t,f)\right\|}_{2}$$

Finally, by inverting the STFT, the time domain of the predicted source signals $\tilde{S}\left(t\right)$ was easily retrieved. In summary, Algorithm 3 describes the source recovery procedure of the technique suggested in of this research.

**Algorithm 3:** Source recovery estimation**Input:** The mixed signal, $\hat{X}\left(t\right)$, and the estimated mixing matrix, $\tilde{A}$.
$\hat{X}\left(t\right)\;is\;transformed$ into the TF domain using STFT.All $M\times \left(M-1\right)$ submatrices of $\tilde{A}$ are placed into A.The source of TF representation is estimated for each TF point.Using inverse STFT, the estimated source signals are transformed back into the time domain.
**Output:** The estimated source signals, $\tilde{S}$.

## 6. Numerical Simulations

In this section, numerical results demonstrating the efficiency of the proposed methods are presented. Taking into consideration the fact that bioacoustic source separation is comparatively understudied in comparison with human speech separation [32], this study addressed the problem of source separation using the bioacoustic mixed signals consisting of four frog species with unique vocal behaviour. Four real bioacoustic signals, $S_{N}(t)$, acquired from the database of [33] were considered. All the signals were recorded in the mono channel at 16-bit resolution, had a 16 kHz sampling rate and were in .wav format. The size of the STFT was set to 1024 in all experiments, the time step was equal to 512, and the weighting function was the Hanning window.

**Figure 3:**
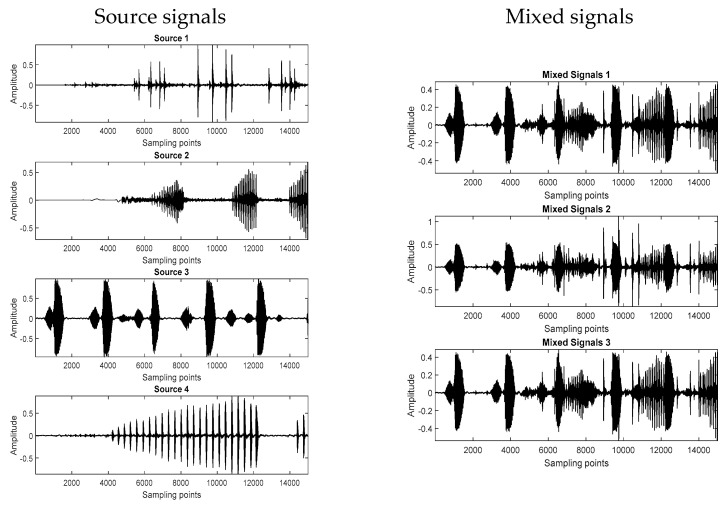
Four original bioacoustic signals and three mixed signals.

In all experiments, the source signal was synthetically mixed to create the mixtures. Four source signals with 15,000 samples were randomly selected and mixed with different random 3 × 4 mixing matrices for each simulation test to generate the mixed signals. For each of the mixture signals, the proposed algorithm was implemented to separate these bioacoustic source signals. The mixed signals were combined with Gaussian noise, and the noise performance of the algorithm at various signal-to-noise ratios (SNR) ranging from 5 dB to 45 dB was tested. The average of 100 Monte Carlo simulation tests was obtained to evaluate the performance of the proposed method. Figure 3 shows the four original bioacoustics signals and the three mixed signal waveforms produced when the SNR was 45 dB.

**Figure 4:**
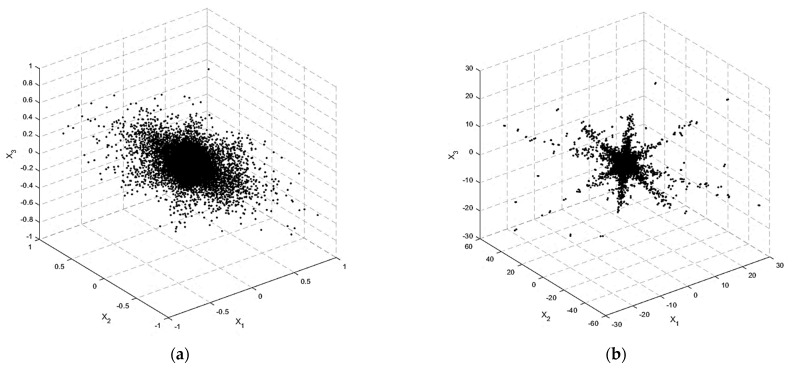
Scatterplot of mixed signals for a scenario with three sensors (M = 3) and four sources (N = 4) of bioacoustic signals. (**a**) Mixtures in the time domain, (**b**) Mixtures in the time–frequency domain.

Natural signals such as bioacoustics signals are not often extremely sparse in the temporal domain. The source signals were made sparser by applying STFT. Figure 4a,b demonstrates the scatterplots of the underdetermined mixtures of three mixtures and four sources of bioacoustics signal in the time domain and TF domain, respectively. The three coordinates in the figures are the three components of the mixed vector, i.e., $X_{1}$, $X_{2}$ and $X_{3}$. Figure 4a shows that the scattered points in the time domain are disorganised, and these points provided limited insight about the mixing matrix. Because of the moderate sparsity of the sources, the directions of the mixtures were obscure. STFT was applied and the column orientations of the mixing matrix became visible in the scatterplot, as shown in Figure 4b. It is clear from the diagram that there were four clumping lines that corresponded to the column vectors of the mixing matrix.

**Figure 5:**
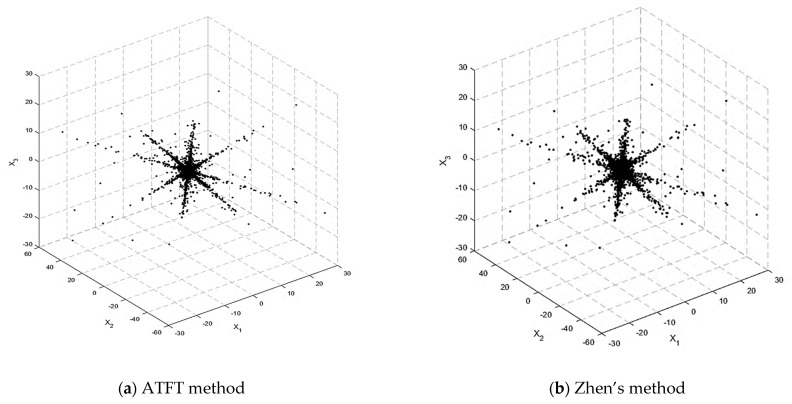
Scatterplot of the TF mixtures’ vectors by (**a**) the ATFT method and (**b**) Zhen’s method.

Figure 5 shows the scatterplot of the TF mixtures after selection by the ATFT and Zhen’s methods. It can be noticed that the ATFT method has a smaller coefficient and a clearer column direction than Zhen’s method. This is because the ATFT method only selects the significant coefficients from the full time–frequency (TF) representation of the vectors of the observed mixed signals. However, Zhen’s method used a fixed parameter to decide which STFT coefficients to use before looking for the SSPs.

**Figure 6:**
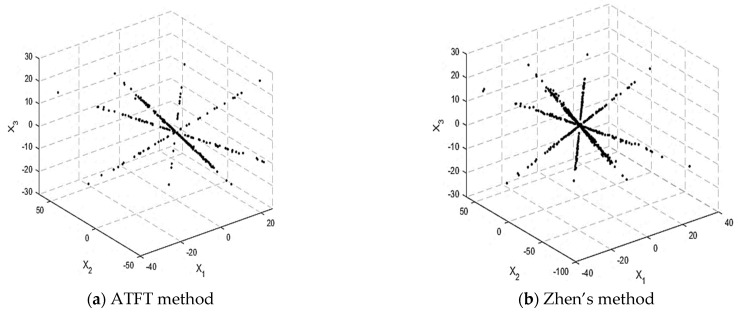
Scatterplot after SSP detection: (**a**) ATFT method and (**b**) Zhen’s method.

Figure 6 depicts the scatterplot following SSP detection using the ATFT and Zhen’s method. There are four lines visible, indicating that the number of sources is four, and their orientation is more distinct than in Figure 5. If we compare Figure 6a,b, it can be observed that the SSP detected in the TF mixtures’ vectors selected by the ATFT method had fewer data points than in Zhen’s method. This result shows that more outliers were effectively removed, indicating the ability of the ATFT method to generate better results than Zhen’s method.

The performance of the proposed technique was assessed by two categories of assessment metrics.

### 6.1. The Performance of Mixing Matrix Estimation

**Figure 7:**
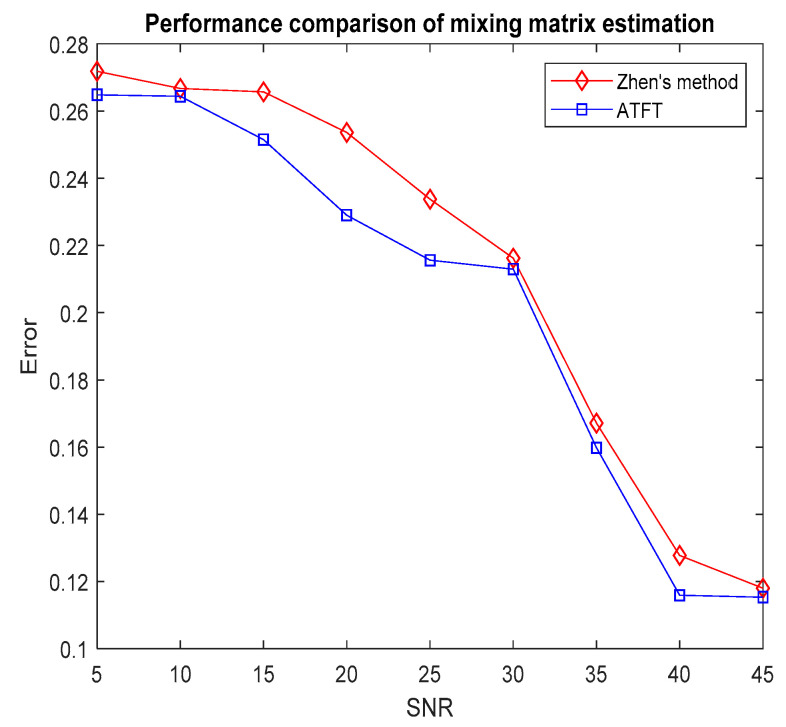
Comparison of the performance of mixing matrix estimation using the ATFT method and Zhen’s method, tested on bioacoustic signals.

The following performance indicator was used in this work to assess the performance of mixing matrix estimation:

(18) $$Error=\frac{1}{n}\sum_{i=1}^{n}\left(1-\frac{{a_{i}}^{T}{\hat{a}}_{i}}{\left\|a_{i}\right\|\left\|{\hat{a}}_{i}\right\|}\right)$$

where ${\hat{a}}_{i}$ denotes the estimation of the mixing vector $a_{i}$ and n stands for the number of sources. In general, as the error reduces, the accuracy of the mixing matrix estimation increases. To assess the efficacy of the investigated technique, the error rate was used to assess the accuracy of the mixing matrix estimation and compare it to Zhen’s method. Figure 7 illustrates the average error obtained after 100 Monte Carlo trials had been run with the ATFT method and Zhen’s method when the SNR ranged from 5 dB to 45 dB.

The simulation results showed that for each method, the accuracy improved with an increase in the SNR. Furthermore, the tests revealed that the ATFT approach was able to predict the mixing matrix more accurately than the existing method. Because of the adaptive threshold setting process, it was able to automatically adapt to the data and consistently achieved accurate estimations. The proposed technique provides an excellent alternative for mixing matrix estimation over the baseline techniques, thus lowering the errors. To analyse the complexity of the research method, the time cost was used to represent the total time in second (s) taken by the CPU for estimating the mixing matrix. The simulations were performed using MATLAB 2016(a) and tested in the Windows 7 operating system with an Intel Core i7 with a 2.93 GHz CPU and 8 GB of RAM. Table 2 shows the time cost of estimating the mixing matrix after 100 Monte Carlo trials of ATFT and Zhen’s method. 

The results showed that the ATFT method was slightly faster than Zhen’s method for all sets in the bioacoustic database, including the speech signals. Zhen’s technique takes the sparse linear representation of the relationships among all time–frequency (TF) mixing vectors into account to determine the locations of the single sources (SSPs). In contrast, the ATFT method uses only the most significant mixtures. Because of the computational difficulty of the approach, the size of the mixture vectors in the ATFT method were sufficient for obtaining an accurate estimation of the mixing matrix. Therefore, the ATFT method cost less than Zhen’s method, since the number of samples needed to estimate the mixing matrix was smaller than in Zhen’s method. In conclusion, the ATFT method provides a more efficient and lower time cost, and it performs better in real-time applications than Zhen’s method.

**Figure 8:**
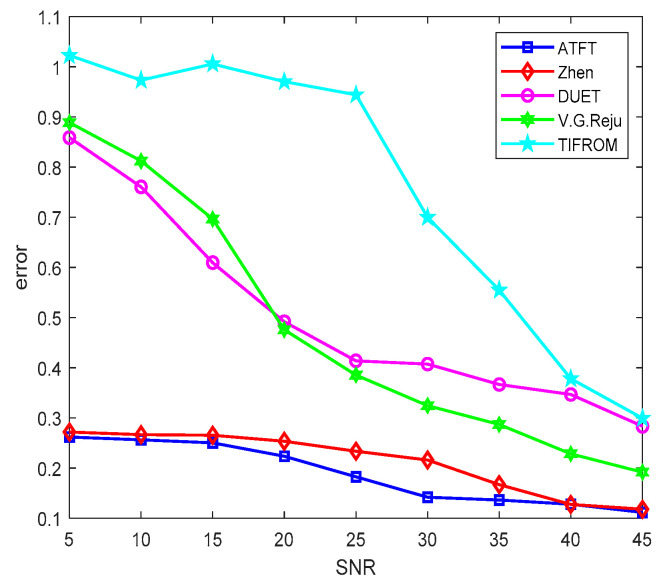
Comparison of the performance of mixing matrix estimation by different methods.

To further validate the relevance of the ATFT method, additional related techniques were also used to demonstrate the performance of the suggested method. The results of [25,34,35] were used to make a quantitative comparison in estimating the mixing matrix. Figure 8 presents the comparative performance of different methods in estimating the mixing matrix against different signal-to-noise ratios (SNR) after 100 Monte Carlo trials for bioacoustic signals.

When the SNR was less than 30 dB, the difference in estimation performance between the methods of DUET, TIFROM, Reju, and Zhen, and the ATFT method was rather large. When the SNR exceeded 30 dB, the performance of all estimation methods reduced. Both the ATFT and Zhen’s methods had a more stable and reliable performance. When the error changed to be not more than 0.2 dB, as the SNR decreased from 45 dB to 5 dB, it was observed that the ATFT method still performed the best. In the noisy case, the ATFT method outperformed the methods developed by [25,34,35] with more than a 0.5 dB difference in the error when the SNR decreased from 45 dB to 5 dB.

**Figure 9:**
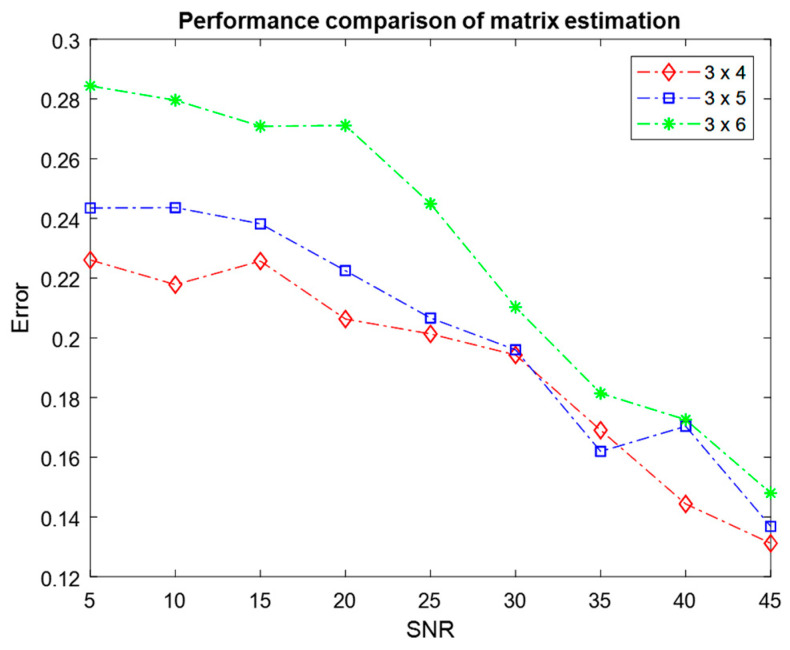
Comparison of the performance of mixing matrix estimation for three mixtures with different numbers of sources using the ATFT method.

**Figure 10:**
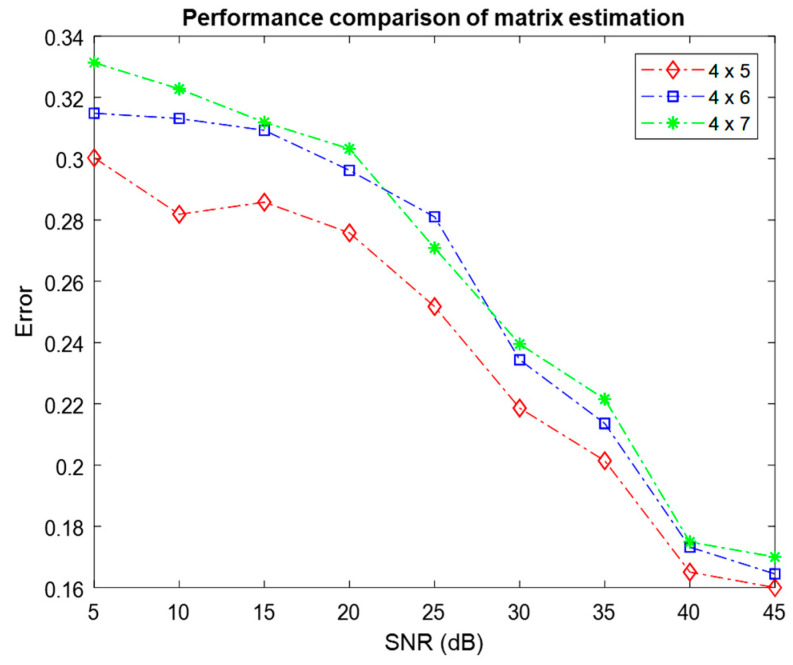
Comparison of the performance of mixing matrix estimation for four mixtures with different numbers of sources using the ATFT method.

The performance of the ATFT algorithm was further evaluated by setting different numbers of mixtures M and different numbers of sources N, where M×N=3×4,3×5,3×6,4×5,4×6,4×7. Figure 9 and Figure 10 depict the mean error resulting from 100 Monte Carlo simulations used to estimate the mixing matrix for three and four mixtures with varying numbers of sources, respectively.

When the performance of different settings is compared between Figure 9 and Figure 10, the setting with three mixtures has smaller average errors at 45 dB SNR. This demonstrated that the setting with three mixtures outperformed the setting with four mixtures in terms of the quality of the mixing matrix estimation. This result demonstrated that the estimation of the mixing matrix for three mixtures was superior to the estimation for four mixtures. The two figures demonstrated that the performance of various sensor setups declined as the number of sources increased. This finding showed a significant correlation between the number of sources and sensors, and the performance.

### 6.2. The Performance of Source Recovery Estimation

The performance of the source recovery estimation was examined using the following method to accomplish bioacoustic source recovery:

(19) $$MSE=10{log}_{10}(\frac{1}{n}\sum_{i=1}^{n}\underset{\delta }{\min}\frac{\left\|{s^{\prime }}_{i}-\delta {\hat{s^{\prime }}}_{i}\right\|\begin{array}{c} 2\\ 2 \end{array}}{\left\|{s^{\prime }}_{i}\right\|\begin{array}{c} 2\\ 2 \end{array}})$$

where ${\hat{s^{\prime }}}_{i}$ denotes the estimation of the source signals ${s^{\prime }}_{i}$ and δ is a scalar reflecting the scalar ambiguity.

**Figure 11:**
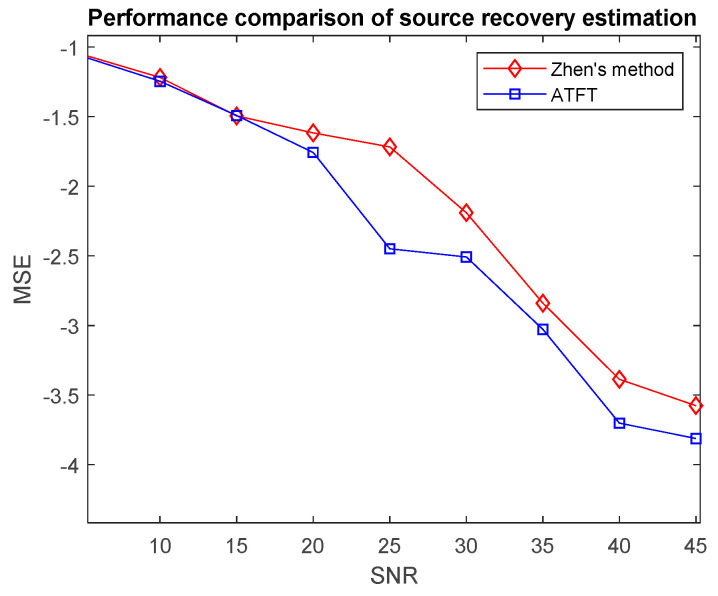
Comparison of the performance of source recovery estimation by the ATFT method and Zhen’s method on bioacoustic signals.

Figure 11 depicts the average source recovery performance over 100 Monte Carlo trials against SNR for the ATFT method and Zhen’s method. This simulation used a 3 × 4 mixing model and the estimated mixing matrix from Section 6.1 to obtain the estimated source by inserting them into the source recovery algorithm. It was discovered that as the SNR increased, the MSE for both methods decreased. This inversely proportional relationship occurred because the noise term diminishes with a higher SNR. A smaller MSE indicates superior quality. The first step of SCA is estimation of the mixing matrix. This step is important because it is required to recover the source signals accurately and will directly affect the second stage. Since the ATFT technique outperformed Zhen’s method in predicting the mixing matrix in Section 6.1, it was demonstrated that the proposed method consistently outperformed Zhen’s method for all levels of SNR.

**Figure 12:**
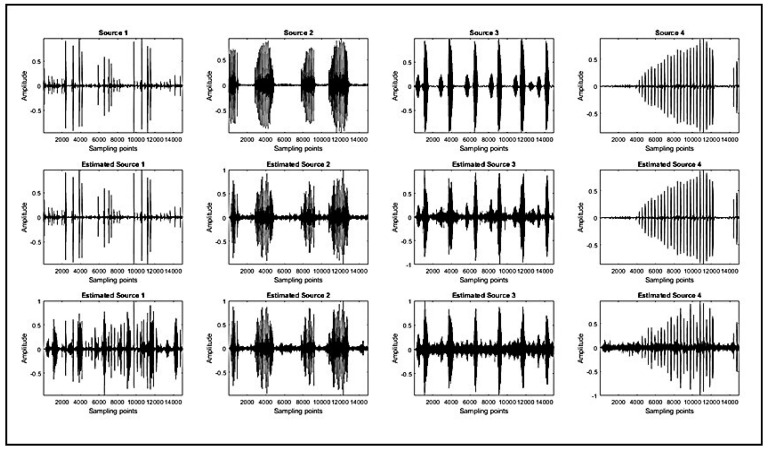
Simulation results of source recovery. First row: the original source signals of four bioacoustic signals. Second row: the estimated sources using the ATFT method. Third row: the estimated sources using Zhen’s method.

The simulation results of the original sources and the estimated sources are shown in Figure 12. The first-row plots are the original sources. The second-row plots represent the estimated sources using the ATFT method. The third-row plots represent the estimated sources using Zhen’s method. The estimated sources obtained using the ATFT method are rather similar to the original sources in comparison with those of Zhen’s method. Despite both the ATFT method and Zhen’s method using the same algorithm for source recovery, the proposed method generated significantly better results. In conclusion, the accuracy of source recovery depends on the estimation accuracy of the mixing matrix.

## 7. Conclusions

The primary objectives of this work were to develop an adaptive algorithm that selected significant coefficients from time–frequency (TF) mixtures to promote better detection of SSPs for estimating the mixing matrix by using the SCA-based UBSS technique. In comparing the proposed method with the benchmark method and several state-of-the-art methods after 100 Monte Carlo trials, the results revealed that the TF selection factor of the proposed method provided higher accuracy than the others for estimating the mixing matrix under different signal-to-noise ratios (SNR) ranging from 5 to 45 dB. In terms of source recovery at all levels of SNR, the proposed method excelled in comparison with the benchmark method approach because of its the more accurate estimation of the mixing matrix that it provided. The simulation results illustrated that the estimated sources were recovered successfully for bioacoustic signals. In conclusion, a method based on the SCA technique has successfully been developed to estimate the mixing matrix and source recovery, and it can address the problems concerning the accuracy, time costs and separation quality of UBSS.

## Figures and Tables

**Table 1:** Summary of research related to the detection of single-source points.

References	Mixing System	Single-Source Point Detection	Limitations
[25]	Two mixtures of three sources from two guitars and one voice	Time–frequency ratio of the mixtures (TIFROM)	Low estimation accuracy for noisy and insufficiently sparse sources
[24]	Three, four and five mixtures each for four to seven speech utterances	Compared the absolute directions of the real and imaginary parts of the TF points in the mixing signals	Limited application, requires real-valued entries in the mixing matrix
[26]	Three mixtures of four sets of sources consisting of the genres of music, speech, instruments and various sounds	An SSD algorithm that recognises the TF points occupied by a single source for each source.	Loses efficiency when the mixing matrix is complex and not real
[27]	Two mixtures of three speech signals	Extracts prior information from the complex-valued mixing matrix at the receiver’s end	Too much computation or poor robustness
[16]	Three mixtures of four speech sources	Sparse coding	Has a fixed parameter to select the STFT coefficients before SSP detection
[28]	Two mixtures of four speech signals	An SSP detection technique based on the transformation matrix	The selection of the peak value used to determine the number of source signals is greatly affected by noise
[29]	Two mixtures of two sets of sources consisting of three male and female speech signals	Calculates the mixing ratio	Sensitive to noise in real-world systems
[13]	Three mixtures of six flutes	Calculating the mixing ratio	Sensitive to noise

**Table 2:** The time cost of estimating the mixing matrix.

Database	Zhen’s Method	ATFT Method
Bioacoustic signals	2.3321	1.9218

## Data Availability

Not applicable.
